# Screening Protein Prognostic Biomarkers for Stomach Adenocarcinoma Based on The Cancer Proteome Atlas

**DOI:** 10.3389/fonc.2022.901182

**Published:** 2022-04-28

**Authors:** Guo-Liang Zheng, Guo-Jun Zhang, Yan Zhao, Zhi-chao Zheng

**Affiliations:** ^1^ Department of Gastric Surgery, Cancer Hospital of China Medical University (Liaoning Cancer Hospital and Institute), Shenyang, China; ^2^ Department of Pathophysiology, College of Basic Medicine Science, China Medical University, Shenyang, China

**Keywords:** stomach adenocarcinoma, TCGA database, TCPA database, protein, prognosis

## Abstract

The objective was to construct a prognostic risk model of stomach adenocarcinoma (STAD) based on The Cancer Proteome Atlas (TCPA) to search for prognostic biomarkers. Protein data and clinical data on STAD were downloaded from the TCGA database, and differential expressions of proteins between carcinoma and para-carcinoma tissues were screened using the R package. The STAD data were randomly divided into a training set and a testing set in a 1:1 ratio. Subsequently, a linear prognostic risk model of proteins was constructed using Cox regression analysis based on training set data. Based on the scores of the prognostic model, sampled patients were categorized into two groups: a high-risk group and a low-risk group. Using the testing set and the full sample, ROC curves and K-M survival analysis were conducted to measure the predictive power of the prognostic model. The target genes of proteins in the prognostic model were predicted and their biological functions were analyzed. A total of 34 differentially expressed proteins were screened (19 up-regulated, 15 down-regulated). Based on 176 cases in the training set, a prognostic model consisting of three proteins (COLLAGEN VI, CD20, TIGAR) was constructed, with moderate prediction accuracy (AUC=0.719). As shown by the Kaplan-Meier and survival status charts, the overall survival rate of the low-risk group was better than that of the high-risk group. Moreover, a total of 48 target proteins were identified to have predictive power, and the level of proteins in hsa05200 (Pathways in cancer) was the highest. According to the results of the Univariate and multivariate COX analysis, tri-protein was identified as an independent prognostic factor. Therefore, the tri-protein prognostic risk model can be used to predict the likelihood of STAD and guide clinical treatment.

## Introduction

Stomach cancer, a malignancy commonly seen among patients, had over 1 million new cases reported and caused 783,000 deaths in the year of 2018 (1). Domestic and foreign studies on the pathogenesis and risk factors of gastric cancer identify known risk factors to include helicobacter pylori infection, family history of upper cancer, history of gastric resection, smoking and consumption of pickled and fumigated food (2), but pathogenesis has not been fully clarified. In recent years, despite some advances in the diagnosis and treatment of STAD, the timeliness and accuracy of prognosis of patients has improved only slightly (3).

At present, the effective treatment for gastric cancer is still surgery, supplemented by radiotherapy and chemotherapy afterwards. In recent years, molecular targeted drugs have been gradually recognized as potentially effective, especially for patients with advanced STAD. Molecular targeted drugs have been rapidly promoted due to their highly targeted toxicity and low side effects (4–6). However, due to the high heterogeneity of STAD and differences in the mechanism of action of various anti-gastric cancer drugs, efficacy is uneven and the overall therapeutic effect is hardly satisfactory. Therefore, if a more efficient and simpler method of early cancer screening can be explored and developed as soon as possible, and targeted gastric cancer drugs with satisfactory efficacy and small side effects can be developed, the survival time of gastric cancer patients can be prolonged as far as possible while also improving patients’ quality of life. It is also important to study the pathogenesis and progression of STAD to guide treatment and improve prognosis.

Today, reverse phase protein array (RPPA) data on 32 cancer types could be obtained from The Cancer Genome Atlas (TCGA) which is funded by the National Institute of Health (NIH) and available on the Cancer Proteome Atlas (TCPA). In addition, from TCGA, numerous “omics” data of different cancer types and clinical data from tumor samples are now available. With the combined use of RPPA data from TCPA and clinical data from TCGA, tumor patients with poor prognosis have been identified in studies. However, as far as we know, there is no such study on STAD. Therefore, we aimed to construct a protein signature model and evaluate its prognostic power for STAD. This article proposes a new method to identify STAD-related proteins, which is beneficial for the identification of new molecular targets and the choice of effective therapies for patients.

## Methods

### Data Collection

The Cancer Proteome Atlas (TCPA) was used to mine data from patient cases with stomach adenocarcinoma (STAD). Reverse phase protein array (RPPA) data (level 4 data) for STAD was downloaded from TCPA (https://www.tcpaportal.org/tcpa/download.html). This dataset consists of 392 patient cases and measures the response to 218 antibodies. We also downloaded the clinicopathological data on 443 cases of STAD from the TCGA (https://portal.gdc.cancer.gov/). Both RPPA data and clinical data were downloaded on January 21, 2020. Since the data is from TCPA and TCGA, no further approval from the ethics committee was required, but we complied with the relevant regulations on TCPA and TCGA data access and patient privacy protection.

### Establishment and Evaluation of Prognostic Risk Model

In this research, the random method was used to allocate patients with STAD into a training set and a testing set, in a 1:1 ratio. Using the former set, overall survival related proteins were identified using a risk ratio (HR) and univariate Cox regression analysis, with proteins having P<0.05 selected as candidate proteins for biomarkers. The candidate proteins were incorporated into further multivariate Cox regression analysis. The coefficient of each model protein was calculated *via* supervised principal component analysis and important proteins were selected using 10-fold cross-validation to eventually construct a prognostic risk model based on proteins’ expression levels. The prognostic model score was equal to the sum of protein expression values multiplied by the corresponding coefficient. Prognostic score= (β1×expression level of protein 1) + (β2×expression level of protein 2) + …(βn×expression level of protein n).

The protein prognostic model obtained from the training set was also used to predict the prognostic scores of the testing set and the set of patients with gastric cancer. The training set, testing set and the all set were divided into a high-risk group and a low-risk group using the median prognosis score in the training set as the critical value. The existence of survival differences between the high and low risk groups was verified using the Kaplan-Meier survival curve. The predictive power of the prognostic risk model was assessed using a Time-dependent ROC curve. The prognostic value of the independent prognostic model was evaluated based on the combination of prognostic risk model and clinical parameters as well as the univariate and multivariate Cox survival analysis. Forest maps were used to show Univariate and multivariate Cox survival analysis results.

### Predicting the Co-Expression of The Three Proteins in the Model and Performing Biological Function Analysis

With a correlation>0.4 and P value<0.001 as screening conditions, 48 proteins related to proteins in the model were found. The Sankey diagram was drawn by the ggalluvial package of R software. In addition, for visual functional analysis, GO and KEGG analyses were performed using Metascape (http://metascape.org/). And P < 0.05 was considered statistically significant.

### Data Processing and Statistical Analysis

The ActivePerl (version 5.26, 64-bit) scripting language was used for the integration and extraction of clinical data. R software (version 3.6.1) and the corresponding R packages were used for data processing and analysis. The extracted clinical data included the sample number, survival time, survival status, age, gender, grade, TNM stage, T status, N status, and M status. The clinical data were merged with the RPPA data. Statistical analyses and data processing were performed using SPSS Statistics 19.0 and R software (version 3.4.4). Volcano map of differentially expressed proteins was generated using the dplyr, ggplot2, and ggrepel software packages. Risk plot, survival plot and heatmap were generated by the pheatmap software package. While the survival curve, univariate and multivariate Cox survival analysis and the ROC curve were generated by survival, survminer, and survival ROC software packages. The mean ± SD was used for the description of continuous variables. The frequency (n) and proportion (%) were used for summarizing categorized variables. The Chi-square test was used for the comparison of proportion. Also, the T test was applied to compare continuous variables, P values are two-sided, and P < 0.05 is considered as statistical significance.

## Results

**Figure 1:**
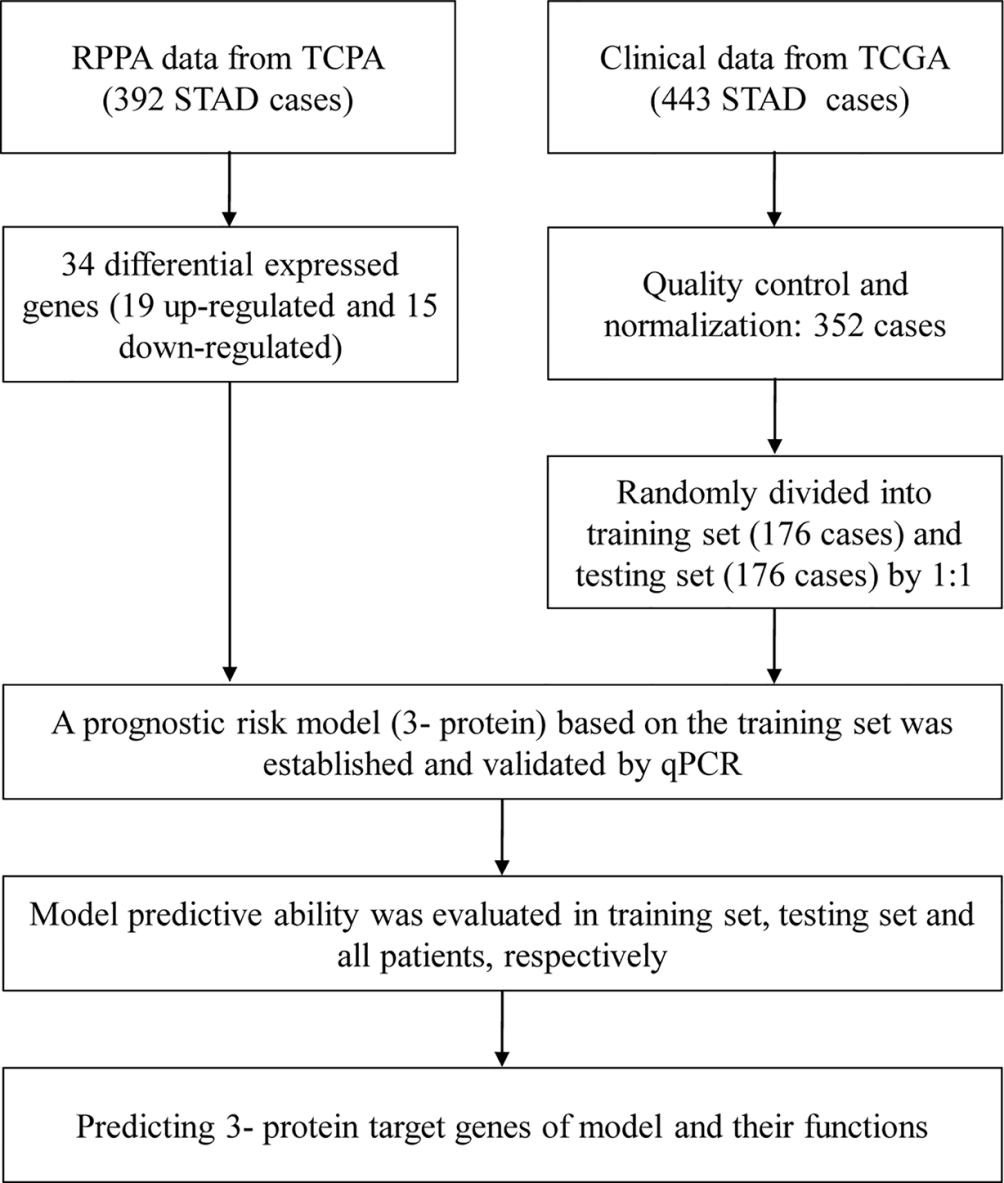
Flow diagram.

In **Figure 1**, we draw a flow diagram to present the workflow more clearly.

### Data of TCGA Protein and Clinicopathological Information of Patients

A total of 352 patients with gastric cancer were finally enrolled into the study, including 222 males and 130 females. The patients were randomly divided into two groups: a training set (n = 176, mean age 65.78 ± 11.03 years) and a testing set (n = 176, mean age 64.72 ± 10.72 years). No significant difference was observed among clinical covariates (P > 0.05) between the two groups, as shown in **Table 1**.

**Table 1:** Clinicopathological data for the training and testing set.

Clinical characteristics	Total (n=352)	Training set (n=176)	Testing set (n=176)	χ2 value	P value
Age (years)	65.25 ± 10.89	65.78 ± 11.03	64.72 ± 10.72	1.059	0.707
Gender				2.391	0.122
male	222	118	104		
female	130	58	72		
Histologic Grade				1.107	0.575
well	7	3	4		
moderate	119	64	55		
poor	226	109	117		
Stage				4.339	0.231
I	39	20	19		
II	106	58	48		
III	167	83	84		
IV	40	15	25		
T				3.764	0.288
T1	12	9	3		
T2	69	36	33		
T3	169	84	85		
T4	102	47	55		
N				1.365	0.243
N0	104	47	57		
N+	248	129	119		
M				3.249	0.071
M0	325	167	158		
M1	27	9	18		

### Screening for Differentially Expressed Proteins

**Figure 2:**
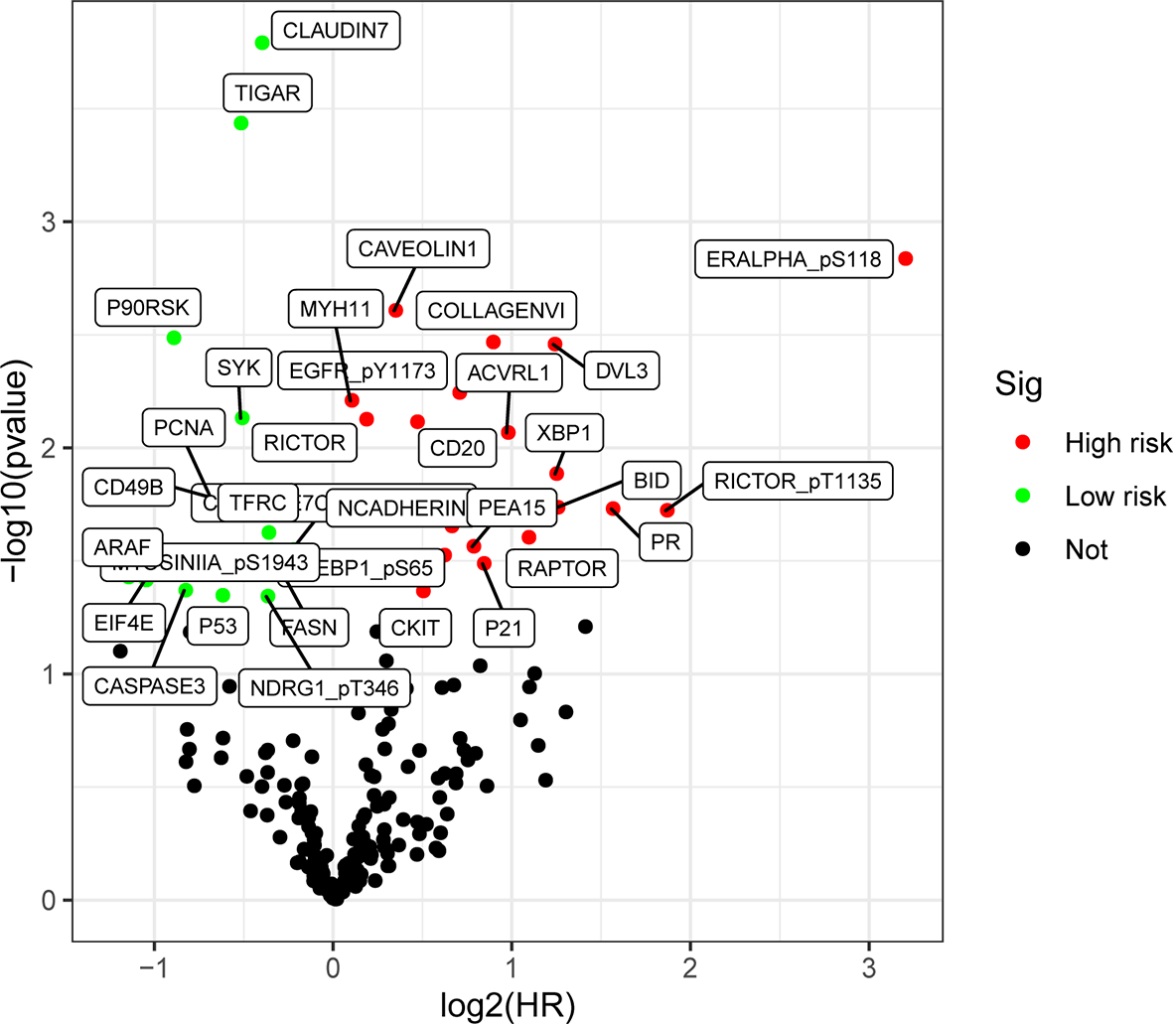
Differential expression of proteins volcano map in gastric cancer. There is a difference in the expressions of up-regulated proteins (HR >1, P< 0.05) (highlighted in red), and down-regulated proteins (HR <1, P < 0.05) (highlighted in green).

The screening criterion was log2 (HR) >1 and P value < 0.05. As shown in **Figure 2**, we analyzed the RPPA data of gastric cancer (n = 392) from TCGA, and screened a total of 34 differentially expressed proteins. Of these, 19 proteins (55.9%) were up-regulated and 15 proteins (44.1%) were down-regulated in gastric cancer.

### Establishment and Evaluation of Prognostic Risk Models

We further conducted univariate and multivariate Cox regression analysis of the training set to establish a prognostic risk model composed of three proteins.

Prognostic risk score = (0.562 × COLLAGEN VI expression level) + (0.499 × CD20 expression level) + (-0.321 × TIGAR expression level).

As shown in **Table 2**, two proteins (COLLAGEN VI, CD20, HR value > 1) were associated with high risk (high expression increased risk of death of the patient), and one protein (TIGAR, HR value <1) was protective (high expression decreased risk of death of the patient). We categorized the cases of the training set into low-risk and high-risk groups based on the median of risk score (score=0.937).

**Table 2:** Prognostic risk model constructed by R language.

Name	Coef	HR	HR.95L	HR.95H	P value
COLLAGEN VI	0.562	1.755	0.951	3.237	0.071
CD20	0.499	1.648	1.128	2.407	0.009
TIGAR	-0.321	0.725	0.525	1.002	0.051

Coef, regression coefficient; HR, risk ratio.

**Figure 3:**
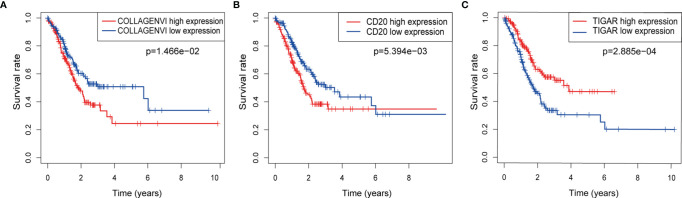
The respective K-M survival prediction curves of the three proteins. **(A)** COLLAGEN VI; **(B)** CD20; **(C)** TIGAR.

**Figure 4:**
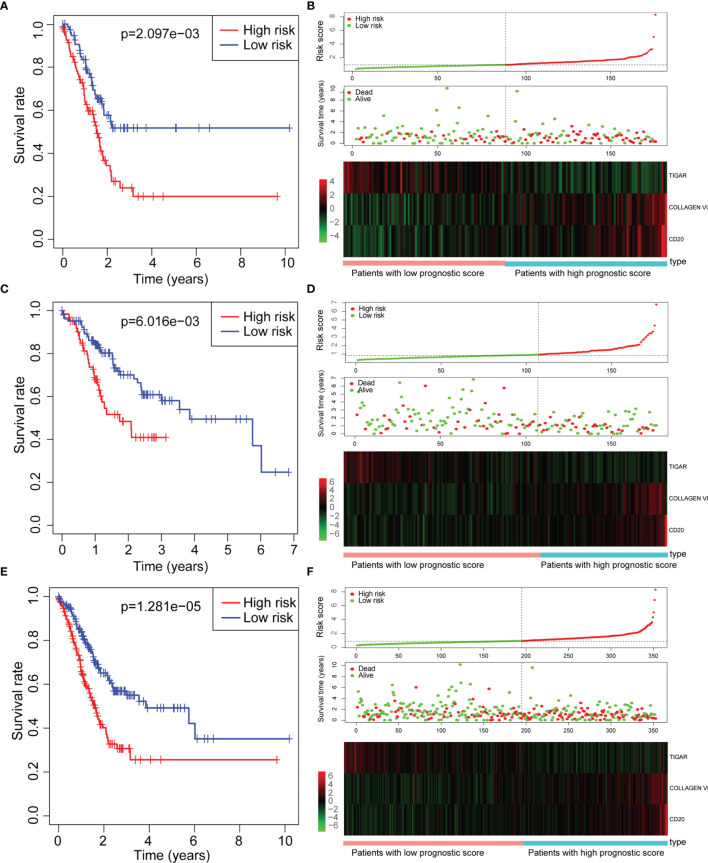
Assessment of prognostic risk models. **(A, B)** Training set; **(C, D)** Testing set; **(E, F)** All set. **(A, C, E)** Kaplan-meier survival curve; **(B, D, F)** Risk score, scattered plots of survival time, and heat map of related proteins expression Note: Abscissa indicates cumulative frequency, and ordinate represents survival time (month). The green circle represents those alive, and the blue diamond represents deaths. The single inflection point of the risk score curve is marked by the dotted line. The gastric cancer patients were categorized into two groups: low risk group and high risk group.

We first performed K-M survival analysis on these three proteins, and found that all three proteins were related to survival (**Figures 3A–C**). Further, we analyzed the risk model of the three proteins. From **Figure 4**, for the training set, the kaplan-meier curve and log-rank test showed a high risk score was associated with poor prognosis (P=2.079×10^-3^) (**Figure 4A**). Cases with high risk score tended to express high-risk proteins, while cases with low risk score tended to express protective proteins. Compared with cases in the low-risk group, those in the high-risk group were observed to have higher mortality. The conclusion was based on the analyses that the lifetime mortality was 29.89% (26/87) in the low-risk group and 55.06% (49/89) in the high-risk group (χ2=11.4, P=0.0007) (**Figure 4B**). The analysis on the testing set and all patients showed similar results (**Figures 4C–F**).

**Figure 5:**
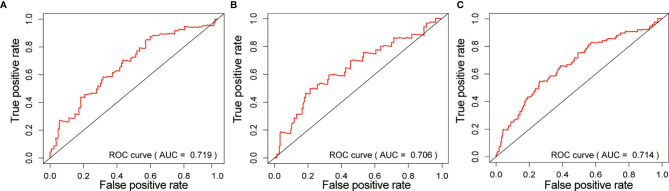
Risk prognostic model in ROC curve of three groups. **(A)** Training set; **(B)** Testing set; **(C)** All set. the abscissa represents the true positive rate (sensitivity), and the ordinate represents the false positive rate (1-specificity).

We also obtained the area under the curve (AUC) of the risk prognostic model for the 3-year survival rate of gastric cancer patients in the training set (AUC=0.719), testing set (AUC=0.706) and all set (AUC=0.714). The AUC values were higher than 0.7, indicating that the model had good prognostic performance, as shown in **Figure 5**, and suggesting that this tri-protein model can be used to predict survival in patients.

**Figure 6:**
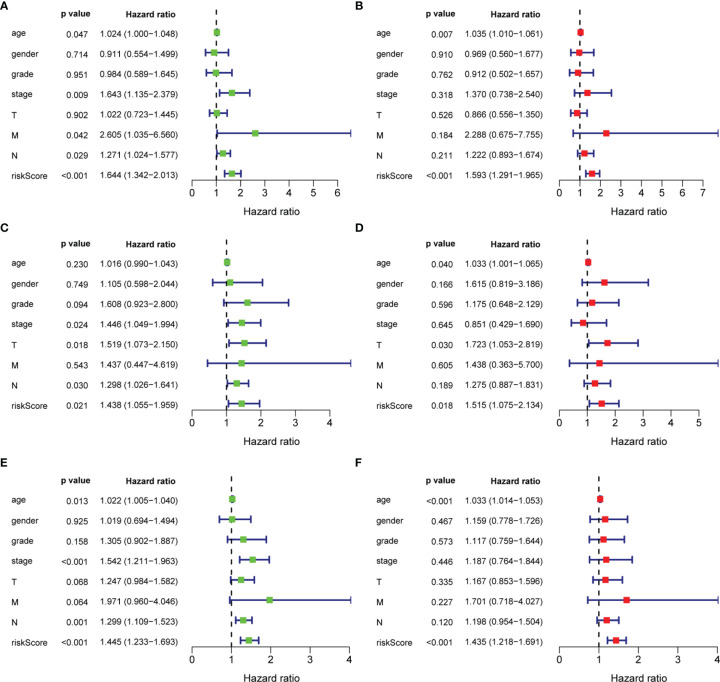
Univariate and multivariate analysis of overall survival of patients. **(A, C, E)** Univariate analysis; **(B, D, F)** Multivariate analysis.

As shown in **Figure 6**, in the training set, there was a significant correlation between age, TNM stage, N status, M status and the (tri-protein) model risk score with prognosis (P<0.05) (**Figures 6A, B**), based on univariate Cox regression analysis. Furthermore, the (tri-protein) model risk score was found to be an independent prognostic factor for STAD (HR = 1.593, P <0.001) by using multivariate Cox regression analysis. The analysis on the testing set and all patients showed similar results (**Figures 6C–F**).

### Analysis of Co-Expressed Proteins and Functions

**Figure 7:**
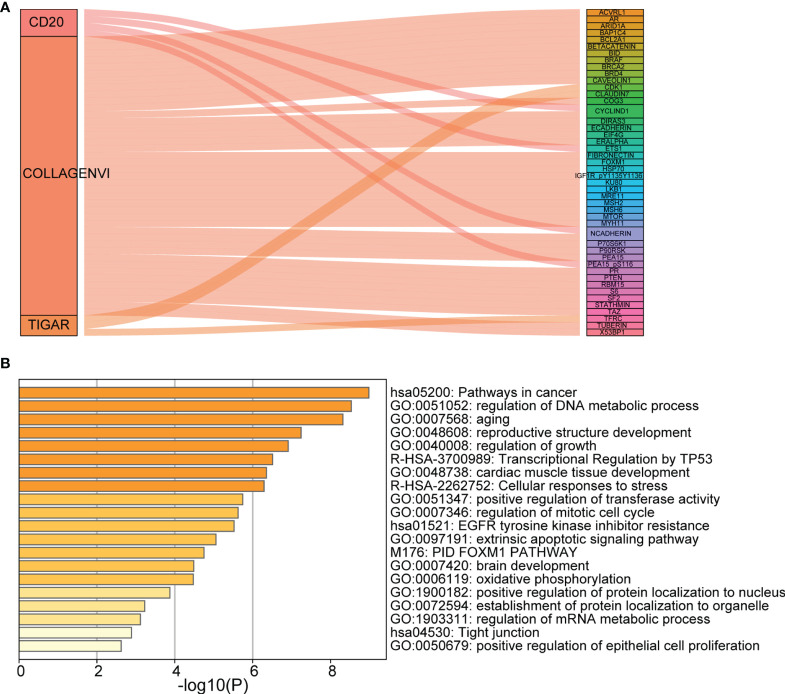
Analysis of co-expressed proteins and functions. **(A)** Sankey diagram; **(B)** The main enrichment pathways (GO and KEGG analyses). Each rectangle denotes a gene, with the connection degree visualized by the rectangle area. The circle area reflects the number of genes on the pathway, while the color depth shows the -log10 (FDR) value. GO, Gene Ontology; KEGG, Kyoto Encyclopedia of Genes and Genomes.

The Sankey diagram of 48 proteins with strong correlation with the three proteins in the risk model was constructed (**Figure 7A**). As shown in **Figure 7B**, the most important enrichment pathways are shown on the Metascape site (GO and KEGG analyses). We found target genes were enriched, mainly in hsa05200: Pathways in cancer, regulation of DNA metabolic process, and regulation of growth.

## Discussion

In this study, proteins differentially expressed in TCPA were screened by bioinformatics technology. Then, the differentially expressed proteins were integrated with clinical parameters to establish a prognostic risk model composed of three proteins. The model showed good prognostic performance in the training set, testing set and all patients (the AUC of the ROC curve predicting 3-year survival was greater than 0.7). More importantly, the multivariate Cox regression analysis further demonstrated that it is an independent factor affecting the prognosis of STAD, so this Tri- protein can be used as a biomarker for the prediction of OS in STAD patients. The target genes and target gene enrichment pathways were predicted to be mostly related to cancer, among which hsa05200: Pathways in cancer has been reported to be involved in the development and progression of gastric cancer. This further indicates that the tri-protein model has a potential role in the molecular pathogenesis, clinical progress and gastric cancer prognosis, and that it is likely to provide help for the preventive diagnosis and individualized treatment of gastric cancer.

Two of the three proteins were high risk factors (expression level was negatively correlated with OS), including COLLAGEN VI and CD20. Originally, COLLAGEN VI was proposed as an extracellular matrix protein, forming a microfilament network and binding to extracellular matrix proteins through the functional subdomains. This is of great importance for organizing fibrillar collagens and being adhesive to the basement membrane (7). In COLLAGEN VI, there are three distinct α-chains (α-1, -2 and -3) and collagen VI α-3 (COL6A3) encodes the α-3 chain, which is longer compared with the other two chains (8). At present, it is widely believed that Collagen VI plays a role in breast and ovarian cancers, arousing the interest of researchers (9–11). There are few reports on gastric cancer at present, and only one literature suggests that COL6A3 may be an oncogene of human gastric cancer, and the antagonism of COL6A3 may be an effective method to treat gastric cancer (12).

As a transmembrane highly hydrophobic glycosylated phosphor protein of 35 kDa, the CD20 protein is encoded in humans by the MS4A1 gene (13). The CD20 protein features in the regulation and differentiation and growth of B cells based on cell activation from the resting state (G0) to the activated state (G1), and regulating cell cycle step-by-step progress from the S phase to mitosis (13). Actually, it is a portion of a cell-surface complex which regulates calcium transport and initiates an intracellular signaling pathway by calcium influx (14, 15). However, critical effects either on B-cell development or immune response implementation (15) have not been shown by disrupting calcium channel gene encoding. In the initial pro-B phase, CD20 has been observed within healthy mature B cells, chronic lymphocytic leukemia, LPHL and classical HL of some patients (14, 16). Moreover, it has also been found that expression of CD20 is also very important in HL disease pathophysiology and is likely to influence the patients,treatment prognosis, relapse and refractory response (16).

One of the three proteins in the developed mode, TIGAR, was low-risk (expression level is positively correlated with OS). TIGAR is a downstream regulator of p53, playing an essential function in metabolism through inhibition of glycolysis and promotion of the pentose phosphate pathway to function oxidative resistance and antiapoptosis (17). Therefore, researchers are more interested in the role of TIGAR in cancer due to its function in glycolysis and redox balance. More and more investigations have been conducted in this field, and they indicate high levels of TIGAR in hematopathy and solid tumors, including acute myeloid leukemia (18), lung cancer (19), colon cancer (20), pancreatic cancer (21) and breast cancer (22). Moreover, high TIGAR expression was an independent predictor of poor survival. Currently, the specific mechanism of the TIGAR is poorly studied, and its relationship with gastric cancer also needs further study. It is hoped that with in-depth study, its biological role and potential mechanism will become clearer.

According to ROC curve analysis, the AUC of the tri-protein model risk score prognosis was greater than 0.7 (between 0.7 and 0.9) in both the training group and the testing group, indicating that the prognostic model had certain accuracy in the diagnosis of STAD. Univariate and multivariate Cox regression analysis further revealed that the risk score model (3- protein) was an independent prognostic factor associated with OS (HR = 1.971, P <0.001). However, there are some shortcomings in this study. Firstly, the data randomly assigned to the training set and the testing set came from a single database. In future studies, setting up a separate set of external tests would make the model’s results more convincing. Secondly, the follow-up time for the TCGA STAD study cohort is relatively short (the average follow-up time was only 20.78 months) and the deletion rate relatively high, which may affect the reliability of the kaplan-meier method. In the future, it is necessary to recruit more STAD patients and conduct longer follow-up studies to verify the findings of this experiment. In addition, the complex effects and specific mechanisms of these miRNAs need to be further studied.

## Conclusion

In summary, our results suggest that our tri-protein model’s risk score significantly differentiates the prognosis of patients with STAD during training and testing, and predicts 3-year overall survival. Therefore, this model may be a novel biomarker based on protein expression level, which is worthy of further study to determine the relevance of its clinical application.

## Data Availability Statement

The original contributions presented in the study are included in the article/supplementary material. Further inquiries can be directed to the corresponding author.

## Author Contributions

In this research, G-LZ made significant contributions to research conception, design of study. G-LZ and G-JZ collected, analyzed, and interpreted the data. G-LZ wrote the first draft of the manuscript. YZ and Z-cZ wrote sections of the manuscript and provided critical revisions. All authors agree to be accountable for all aspects of the work, and questions relevant to accuracy or integrity are dealt with and surveyed in an appropriate way. The final manuscript has been read and approved by all authors.

## Funding

This study was supported in part by grants from Cultivation fund project of National Natural Science Foundation of China (No. 2021-ZLLH-06) and Medical engineering cross research fund project of Liaoning Province (No. LD202014).

## Conflict of Interest

The authors declare that the research was conducted in the absence of any commercial or financial relationships that could be construed as a potential conflict of interest.

## Publisher’s Note

All claims expressed in this article are solely those of the authors and do not necessarily represent those of their affiliated organizations, or those of the publisher, the editors and the reviewers. Any product that may be evaluated in this article, or claim that may be made by its manufacturer, is not guaranteed or endorsed by the publisher.

## Abbreviations

AUC, Area Under The Curve; Coef, Regression coefficient; FDR, False Discovery Rate; GO, Gene Ontology; HR, Hazard Ratio; KEGG, Kyoto Encyclopedia Of Genes And Genomes; LogFC, Log2 Fold Change; OS, Overall Survival; STAD, Stomach Adenocarcinoma; QPCR, Quantitative Polymerase Chain Reaction; RPPA, Reverse phase protein array; ROC, Receiver Operating Characteristic Curve; TCGA, The Cancer Genome Atlas; TCPA, The Cancer Proteome Atlas.
